# Endosonography-guided suture device for gastrointestinal lumen-to-lumen apposition in a porcine model

**DOI:** 10.1016/j.igie.2024.08.009

**Published:** 2024-08-30

**Authors:** Jad AbiMansour, Shunsuke Kamba, Barham K. Abu Dayyeh, Ryan J. Law, Vinay Chandrasekhara, Elizabeth Rajan, Andrew C. Storm

**Affiliations:** Division of Gastroenterology and Hepatology, Mayo Clinic, Rochester, Minnesota, USA

## Abstract

**Background and Aims:**

The ability to effectively tether 2 separate lumens would simplify performance of transluminal therapeutic endoscopic ultrasound (EUS). This article describes a novel device permitting simple and effective luminal apposition under EUS guidance.

**Methods:**

A porcine preclinical study using 1 domestic pig was performed to show proof-of-concept of a novel suturing device that can be deployed with EUS to appose the gallbladder wall to the stomach. The goal is to facilitate a simple and straightforward approach to cholecystogastrostomy using a lumen-apposing metal stent.

**Results:**

EUS was performed to identify the porcine gallbladder. The device was used to puncture across the gastric wall and into the gallbladder. Once needle access was obtained, 4 flexible braided suture tags were extruded over a 3-0 polypropylene suture into the gallbladder lumen. Upon applying suture tension, the tags bunch to form a secure loop within the gallbladder lumen. With tension on this suture, the gallbladder may be brought up to the gastric wall and the transmural 3-0 suture then cinched at the gastric mucosa to effect durable luminal apposition. After this, cholecystogastrostomy with an electrocautery-enhanced lumen-apposing metal stent was performed using a standard freehand technique.

**Conclusions:**

A novel EUS-guided suturing device permits apposition of the porcine gallbladder and stomach, facilitating EUS-guided transmural gallbladder drainage. This approach may affect the safety and technical success rates of novel translumenal therapeutic EUS procedures.

Endoscopic ultrasound (EUS)–guided drainage of the gallbladder (EUS-GBD) using a lumen-apposing metal stent (LAMS) is now cleared by the U.S. Food and Drug Administration as an endoscopic approach for the management of acute cholecystitis in patients who are not candidates for surgery.^1^ It is one of many therapeutic endoscopic procedures that provides definitive management for conditions that previously required surgical intervention. Advances in therapeutic EUS techniques have led to endoscopic approaches to the drainage of abdominal fluid collections (eg, cystogastrostomy), gastric outlet obstruction (eg, gastroenterostomy), and biliary access in patients with altered anatomy (eg, EUS-directed transgastric endoscopic retrograde cholangiopancreatography). The foundation of these endoscopic techniques relies on apposition of 2 lumens to provide drainage or access. In the case of EUS-GBD, the gallbladder is apposed to the stomach or duodenum using an electrocautery-enhanced LAMS to maintain patency and prevent migration.

Reported outcomes of EUS-GBD show high technical and clinical success rates, ranging from 90% to 98.7% and 89% to 98.4%, respectively.^1^ However, the feasibility of EUS-GBD is dependent on the location of the gallbladder lumen, typically within 10 mm of the enteric or gastric wall, and the ability to manage adverse events should the stent be mal-deployed. Accordingly, performing EUS-GBD is generally limited to high-volume centers with expertise in interventional EUS. The ability to appose the gallbladder lumen to either the stomach or duodenum before use of cutting electrocautery and subsequent advancement of a large-diameter stent deployment catheter into the gallbladder may make the procedure more accessible due to reduction in technical demands and improved safety.

The adverse event rate of EUS-GBD is highly variable among studies; however, one meta-analysis comparing EUS-GBD versus percutaneous drainage reported an adverse event rate of 20%.^2^ Although the majority of adverse events develop in the postprocedural period, the most serious and common intraprocedural adverse events include stent mal-deployment, resulting in a perforation at the site of duodenal or gastric puncture or, in the worst case scenario, also the gallbladder wall. Poor apposition may also lead to issues with pneumoperitoneum, bile leak, and perotinitis.^3^^,^^4^

Data on EUS-GBD learning curves are limited, but unplanned procedural events in one study occurred in 11.3% of cases related to dislodged guidewires or mal-deployment of the stents. These events were distinct from adverse events and more common in patients without acute cholecystitis who had gallbladders that were fibrosed and nondistended as well as in procedures performed by less-experienced endoscopists.^5^ The ability to affix the gallbladder to the stomach or small bowel would theoretically improve the technical demands of the procedure, making it more akin to drainage of a pancreatic fluid collection, which is considered more straightforward and is therefore performed more commonly by therapeutic endosonographers.^6^

No current, clinically available device is able to perform lumen-to-lumen mucosal apposition. Therefore, a proof-of-concept study was performed by using a novel suturing device (SofTac EndoFix, Wrentham, Mass, USA) that is deployed under endoscopic and/or EUS guidance serving to appose 2 tissues to facilitate EUS-GBD.

## Methods

A proof-of-concept, in vivo preclinical study was performed using a single, 45 kg domestic pig. The protocol was approved by the Mayo Clinic Institutional Animal Care and Use Committee and followed American Physiological Society guidelines for the care and use of animals. The animal was started on a liquid diet 48 hours before the procedure, followed by a clear liquid diet 24 hours prior. The pig was kept nil per os on the day of intervention. Induction was performed with intramuscular Telazol 5 mg/kg and xylazine 2 mg/kg, followed by intubation and mechanical ventilation with 2% isoflurane maintenance anesthesia. A linear echoendoscope (EG-3870UTK; Pentax Medical, Tokyo, Japan) was advanced into the porcine stomach, and the gallbladder was visualized. Once the gallbladder was seen, the device was inserted into and advanced from the working channel of the echoendoscope (Video 1, available online at www.igiejournal.org).

The protective sheath was withdrawn, and the 19-gauge needle sharpened via withdrawal of an inner stylet. The needle was used to puncture the gallbladder, which was several centimeters distant to the gastric wall, followed by deployment of three 12.5 mm braided polyester tags strung on a 3-0 polypropylene suture under EUS guidance. The device was then exchanged out over the suture, and gentle traction was applied to bring the gallbladder into close apposition to the gastric wall. Once appropriate positioning was confirmed, a cinching device was passed over the suture. This cinching catheter simultaneously ligates and secures the suture with a plastic cinch resulting in locking of the suture construct to the gastric wall. This process was repeated with a second device to create 2 points of fixation between the gallbladder and gastric wall.

A cholecystogastrostomy was then performed by a trainee endosonographer with <25 cases of experience utilizing an electrocautery-enhanced 15 × 10 mm LAMS (Axios; Boston Scientific, Marlborough, Mass, USA). The stent was advanced while applying pure-cut current at 100 W from an electrosurgical generator (Erbe IC200; Marietta, Ga, USA) and deployed in standard fashion. Technical success was defined as the ability to appose the gallbladder to the gastric wall and successful cholecystogastrostomy formation. The stent was then dilated to 15 mm using a through-the-scope balloon dilator (CRE Balloon; Boston Scientific) to facilitate examination of the gallbladder with a gastroscope (GIF-H190; Olympus Corporation, Center Valley, Pa, USA). The gastric puncture site was monitored for 15 minutes to assess for early separation, gastrointestinal (GI) bleeding, or perforation followed by euthanasia of the animal.

## Results

**Figure 1:**
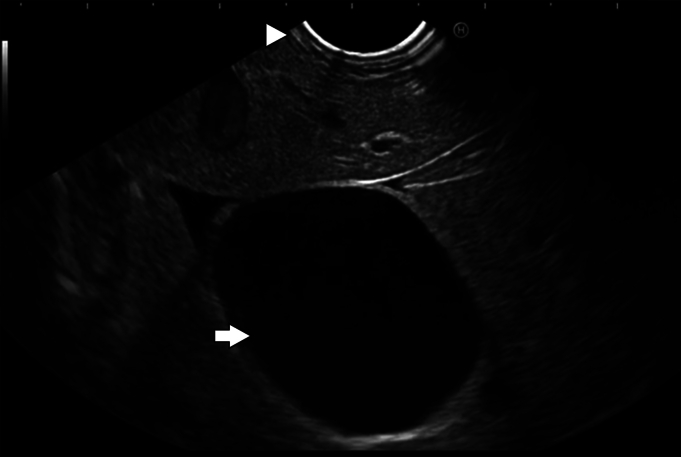
Gallbladder (*arrow*) visualized endosonographically, notably in a location far from the gastric wall (*arrowhead*).

**Figure 2:**
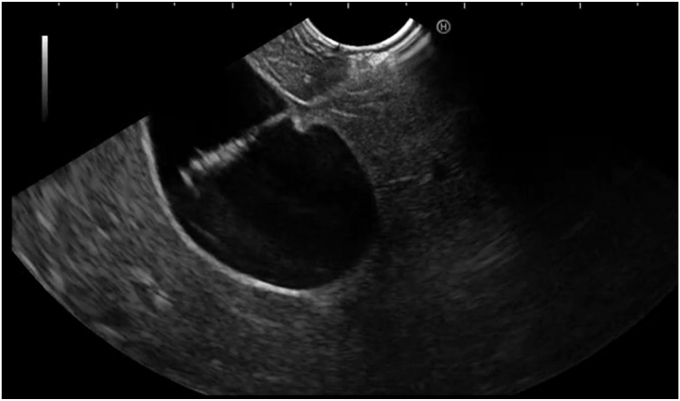
Device was advanced into the gallbladder.

**Figure 3:**
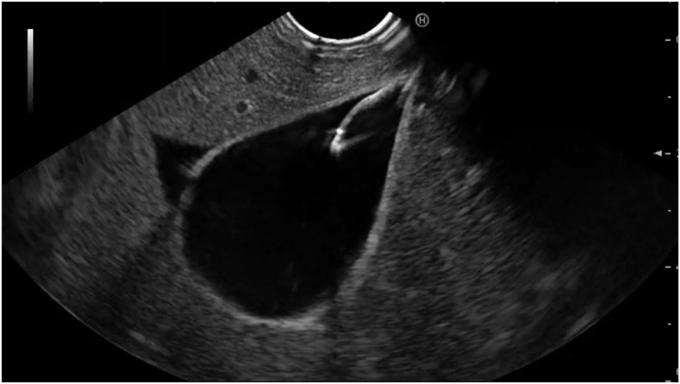
Advancement of securement tags through the device.

**Figure 4:**
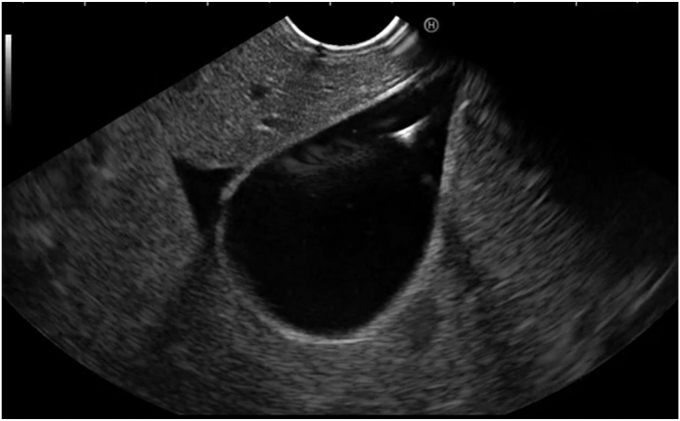
Successful mobilization and traction of the gallbladder.

The gallbladder was successfully visualized endosonographically after careful examination of the stomach, notably in a location several centimeters from the gastric wall in a position that would be relatively contraindicated to standard LAMS cholecystogastrostomy creation otherwise (Fig. 1). The device was advanced into the gallbladder in a fashion similar to standard EUS-guided fine-needle aspiration and biopsy needles (Fig. 2). The braided suture tags were then advanced through the needle and were promptly visualized endosonographically forming a tethering loop (Fig. 3). Tension applied to the suture led to successful mobilization of the gallbladder until it made direct contact with the gastric wall (Fig. 4). Visualization of the gastric puncture site was made before cinch deployment utilizing endoscopic, fluoroscopic, and EUS visualization to confirm final tension and apposition of the gallbladder and bowel walls. The cinch was deployed with gentle traction applied to the suture.

**Figure 5:**
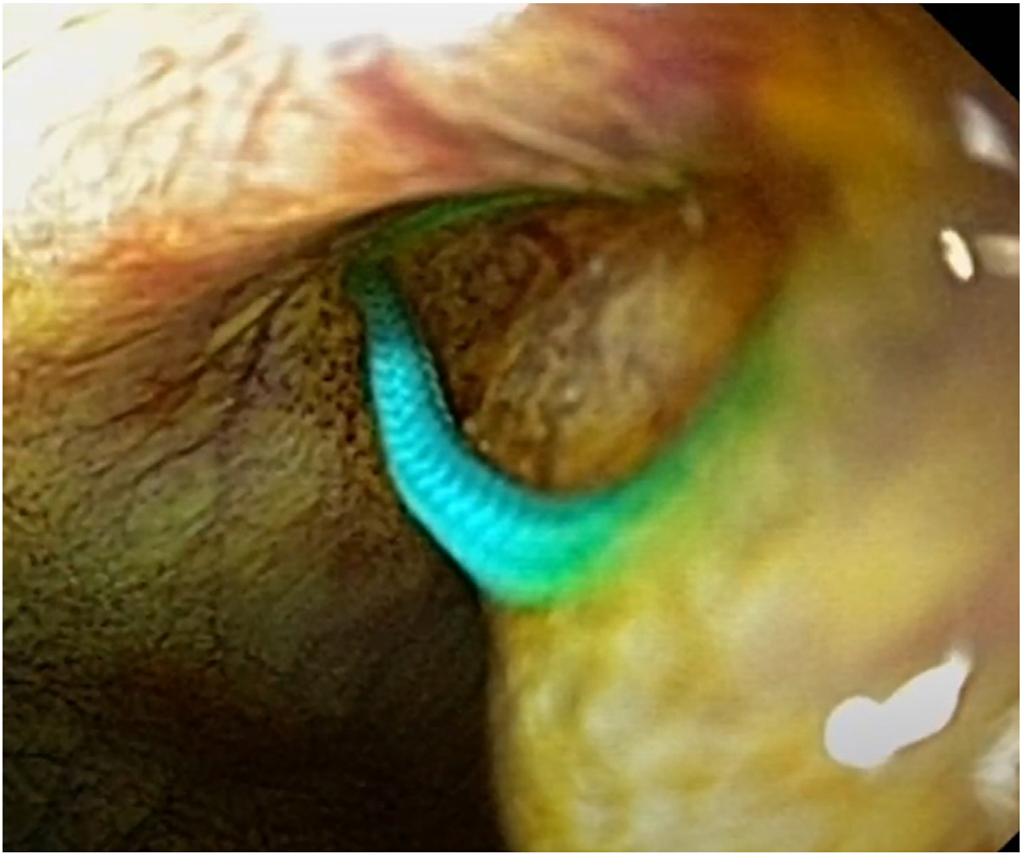
Securement tags visualized from within the gallbladder.

This process was successfully repeated with a second EUS-guided suture device resulting in multi-point fixation of the gallbladder to the gastric wall. This allowed for straightforward creation of a cholecystogastrostomy through deployment of an electrocautery-enhanced LAMS using a freehand technique without intraprocedural adverse events. The procedure was technically successful and there were no immediate adverse events. A gastroscope was advanced through the upper GI tract and through the LAMS to visualize the sutured cholecystopexy from within the gallbladder lumen (Fig. 5). The second fixation point was not visualized endoscopically as it was located behind the distal flange of the LAMS.

## Discussion

From gallbladder drainage to bypassing malignant gastric outlet obstruction, advancements in EUS technologies have driven increasingly complex and invasive therapeutic procedures to accomplish what a decade ago could only be performed surgically or percutaneously. These novel techniques are largely derived from advancements aimed at natural orifice transluminal endoscopic surgery following the paradigm of breaching a hollow viscus wall to enter the peritoneum and perform a surgical intervention.^7^ However, through the use of EUS in modern practice, similar complex surgical tasks may be approached often without entering the peritoneum thanks to advances in EUS platforms and the advent of the LAMS.^8^

Although the barbell shape of the LAMS allows for robust luminal apposition once deployed, their use is inherently limited by patient anatomy and a short 10 or 15 mm stent length. Failure to account for the distance between 2 target GI lumens may lead to adverse events related to stent mal-deployment or perforation, resulting in severe consequences that require complex endoscopic or surgical salvage. The porcine anatomy in this case highlights this point as the initial gallbladder position was not amenable to conventional EUS-GBD using available devices. However, the use of a suture-based luminal apposition device made the procedure relatively straightforward once the gallbladder was repositioned and affixed to the gastric wall, allowing for rapid deployment of a relatively large 15 × 10 mm LAMS by a trainee endosonographer. The use of a 10 × 10 mm LAMS may also be readily considered in this clinical scenario.

There has been significant interest in making EUS-guided novel anastomosis creation procedures safer and more accessible; for example, an overtube-like device was intended to help fix and maintain a small-bowel target for the creation of EUS-guided gastrojejunostomy.^9^ However, these types of devices are procedure specific, requiring additional time and technical knowledge that may not apply to all novel anastomosis procedures. There have been other devices that use modified needles to deploy cross-like stents or sutures to anchor target tissue during flexible endoscopy, but these were largely developed before the widespread adoption of LAMSs.^10^^,^^11^ A more recent report describes the use of suture T-tags to accomplish fixation before endoscopic pancreatic cyst drainage.^12^ However, no commercially available device has the functions necessary to appose 2 lumens before stent deployment. The device used in this study utilized an archetype that is similar to commonplace tissue acquisition needles and can be deployed quickly without the need to change position or the (echo)endoscope operating platform.

The ability to selectively target and appose different lumens in the abdomen has the potential to significantly affect the current interventional EUS paradigm, and the technique described here would be readily applicable to EUS-guided gastrojejunostomy as well as EUS-directed transgastric endoscopic retrograde cholangiopancreatography. In a broad sense, it would allow for endoscopists to “navigate” the peritoneum and manipulate abdominal viscera in ways previously restricted to laparoscopy and continue to perform complex interventions without entering the peritoneum directly, much in the spirit of the natural orifice transluminal endoscopic surgery movement.^7^ It may also reduce the technical demands of these otherwise complex procedures and improve safety, making these procedures more accessible to endoscopists and ultimately to patients.

No adverse events were noted in this proof-of-concept study, but bile leak may be one potential adverse event of gallbladder puncture if gallbladder drainage is ultimately unsuccessful. However, the ability to appose mucosa and cinch the puncture site likely reduces the risk of a clinically significant leak. Other considerations that could potentially limit the use of the device include a scarred, fixed gallbladder or markedly friable tissue, such as with chronic cholecystitis, which would preclude apposition or manipulation of the gallbladder tissue. The target lumen must also be endosonographically accessible with displaceable intervening soft tissue structures.

The current study is limited by its design as a proof-of-concept study that utilized a single animal. Further preclinical and clinical investigation is required to show both efficacy and safety. In addition, although luminal apposition is key to several intervention EUS procedures, success with EUS-GBD does not necessarily translate to other interventions unilaterally. This model utilized a healthy porcine gallbladder. Additional considerations are required for use of the device in diseased gallbladders. Lastly, it is unclear whether the tags would require removal after successful stent placement, and this should be investigated with survival studies. The tags may in theory serve as a nidus for stone and scar formation.

The field of interventional EUS is rapidly expanding, and it is essential that endoscopes, devices, and accessories evolve in parallel to maximize efficacy, efficiency, and safety for patients. The device described in the current study shows the feasibility and relative ease by which the gallbladder may be targeted, maneuvered, and fixed in place during EUS, highlighting a strong potential for future investigation regarding device refinement and applicability to other endoscopic procedures.

## Disclosure

The following authors disclosed financial relationships: S. Kamba: consultant for LPixel and Fujifilm; research grant from 10.13039/501100002424Fujifilm; and royalties from Boston Scientific Japan. V. Chandrasekhara: consultant for Boston Scientific and Covidien; research funding from Micro-tech Endoscopy and STARmed; and stock shareholder of Nevakar Corporation. B.K. Abu Dayyeh: grants/research support from Apollo, 10.13039/100023790Aspire, Cairn Diagnostics, and Spatz; consultant for BFKW, Boston Scientific, Endogenex, EndoGastric Solutions, MetaModix, and USGI; speaking for EndoGastric Solutions, Johnson & Johnson, Medtronic, and Olympus; and teaching for Olympus. R.J. Law: consultant for ConMed, Boston Scientific, and Medtronic. E. Rajan: royalties from Medtronic and Ruhoff Inc; and consultant for Olympus and Johnson & Johnson. A.C. Storm: research grants from Apollo Endosurgery, Boston Scientific, Endogenex, endo-TAGSS, EnteraSense, MGI Medical, OnePass, and SofTac; and consultant for Ambu, Boston Scientific, Intuitive, Medtronic, Microtech, and Olympus. J. AbiMansour: none declared. Funding for this study was provided by SofTac.

## Supplementary data

Video 1 Device overview and case highlighting its use for endosonography-guided gallbladder drainage.
